# Effects of total daily light integral from blue and broad-band red LEDs on flowering of saffron (*Crocus sativus* L.)

**DOI:** 10.1038/s41598-023-34424-0

**Published:** 2023-05-03

**Authors:** Dan Gao, Xinyu Ji, Qing Yuan, Weizhong Pei, Xue Zhang, Fusheng Li, Qiuyi Han, Shanduan Zhang

**Affiliations:** 1 Institute for Electric Light Sources, Fudan University, Shanghai, 200438 China; 2 Shanghai Traditional Chinese Medicine Co Ltd, Shanghai, 200082 China

**Keywords:** Light responses, Photosynthesis, Plant breeding

## Abstract

Present indoor cultivation of saffron (*Crocus sativus* L.) only depends on artificial planting experience, so that flower number and stigma yield are seriously affected in case of cloudy or rainy days and temperature changes. In this study, a luminaire was used at 10-h photoperiod combined 450 nm blue LEDs with 660 nm broad-band red LEDs, which respectively had full width at half maximum (FWHM) of 15 nm and 85 nm, in a ratio of blue: red: far-red light = 20%: 62%: 18%. The influence of total daily light integral (TDLI) was evaluated on flowering characteristics, stigma quality, as well as leaf morphological characteristics. The results showed that flower number, daily flowering proportion, stigma dry weight and crocetin esters content were significantly correlated with TDLI (*P* < 0.01). The increasing TDLI could slightly promote leaf width and leaf area beyond buds, but had no significant effect on bud length and leaf length. Both the average flower number per corm and dried stigma yield was the highest under the 150 mol m^−2^ TDLI treatment, up to 3.63 and 24.19 mg respectively. The former was 0.7 more than that under original natural light treatment, while the later was 50% higher. Totaling, combining blue LEDs with a broad-band red LEDs of the 150 mol m^−2^ TDLI was the most favorable condition for flower number and stigma quality of saffron in this study.

## Introduction

Saffron (*Crocus sativus* L.), a member of the large family Iridaceae, is well known for its uses in spices, dyes, perfumes, and herbal medicine^1–5^. It has been now cultivated in Italy, Spain, Greece, Iran, India, and Afghan, etc^5–7^. After being introducing into China, saffron is mainly cultivated in the Yangtze River Delta with a special seasonal cultivation method. Due to high temperature and humidity in summer in this region, saffron is grown outdoors from late November of the first year to early May of the second year for clonal multiplication and nurtured indoors from late May to early November for blossom^8,9^. There has been increased interest in biological effects and potential medical applications of the long scarlet stigmas of saffron, particularly in menstruation disorders, metabolic syndrome, anti-depression, anticancer and anti-tumor field^10–12^.

However, the yearly yield of saffron stigma is very low, causing short supply and expensive price in the market^6,7,13^. There are two pivotal reasons: (1) Saffron only relies on clonal corm multiplication, and low reproduction rate leads to the difficulty of obtaining high quality corms for propagation^4,14^, (2) Saffron indoor cultivation mainly depends on artificial experiences, and thereby uncontrollable environment conditions further aggravate the phenomenon of less or even no flowering of corms and poor harvest of stigma. Among environmental factors, light is one of the most important variables affecting plant growth and flowering^15^. Several researches on the effects of light spectrum, light intensity and photoperiod on corm reproduction and saffron flowering were conducted to solve the problems aforementioned.

Moradi et al*.*^16^ investigated the effects of different ratios of red and blue light (including 100%, 75%, 50%, 40%, 25% and 0% blue light) on photosynthetic performance, biomass partitioning, as well as morphological and biochemical characteristics of saffron, and concluded that increasing the ratio of blue to red can improve the production of high-quality daughter corms and alter biomass partitioning towards harvestable organs (corms and flowers) in saffron. Zhu et al*.*^17^ found that monochromatic red light LED promoted saffron growth, advanced flowering, and improved total stigma dry weight and crocin production. Kajikawa et al*.*^18^ found that there was no significant difference in shoot length, maximum diameter, weight, and stigma yield of daughter corms when irradiated with two ratios of red light and far-red light (R/FR = 15.8 and R/FR = 1.8), but there was a significant difference in the absorbance of crocetin solutions. It was presumed a lower R/FR ratio at the stage of daughter corms development could induce increase in crocetin. The two aforementioned saffron studies did not present any results on saffron leaves. Under supplemental lighting, Ji et al*.*^8^ measured the spectral response curves of saffron leaves, and found that the peak wavelengths of supplemental lighting spectrum should be at 480 nm and 660 nm, which would benefit the mechanical analysis of spectrum on biomass accumulation. As for photosynthetic characteristics, Renau-Morata et al*.*^4^ showed that the photosynthetic rate was consistently very high (26 μmol m^−2^ s^−1^) throughout the year but was reduced in the largest corms. Meanwhile, the daughter corms were primarily supported by the photosynthesis in the leaves that contributed 90% of the biomass accumulation in the organs of saffron. Koocheki et al*.*^19^ showed that light and temperature conditions had a significant effect on the length of emergence of aerial parts, aerial dry matter, leaf area and number of active shoots on saffron corms, and increasing photoperiod from 6.5 to 16 h increased all these components. Nevertheless, only 33% of corms bloomed when corms were located under light condition of 16 h/8 h (light/dark) compared with 75% for corms located under natural conditions, while corms located under the light condition of 6.5 h/5.5 h (light/dark) did not bloom. Zhu et al.^20^ found that dry weight per stigma was the heaviest under 10 h/14 h treatment, with a significant difference from 14 h/10 h treatment (*P* < 0.05) with the corm weight of 20–25 g.

From the aforementioned studies, it is hard to draw a firm quantitative conclusion on how light affect the flowers and corms of saffron due to different corm provenances, mother corm sizes, planting conditions, cultivation methods, geographical and climatic characteristics and experimental settings^5–7,21,22^. More researches are necessary on artificial lighting of saffron. Moreover, saffron and corm production should be considered as two distinct production processes because the growth conditions maximizing corm and stigma yield are different. Whereas in general, the combination of blue and red light has been considered as the recommended light conditions, but there are some differences in the specific spectral ratio, photoperiod, and light intensity.

The effect of total daily light integral (TDLI) of artificial lighting on saffron growth has not been investigated so far. The aim of the present study is to use a certain ratio of mixed blue and red light and to characterize the influence of different TDLI on saffron flowering, stigma quality and corm leaves indoors to provide a guiding direction for stigma yield improvements.

## Materials and methods

### Plant material and growth conditions

**Figure 1:**
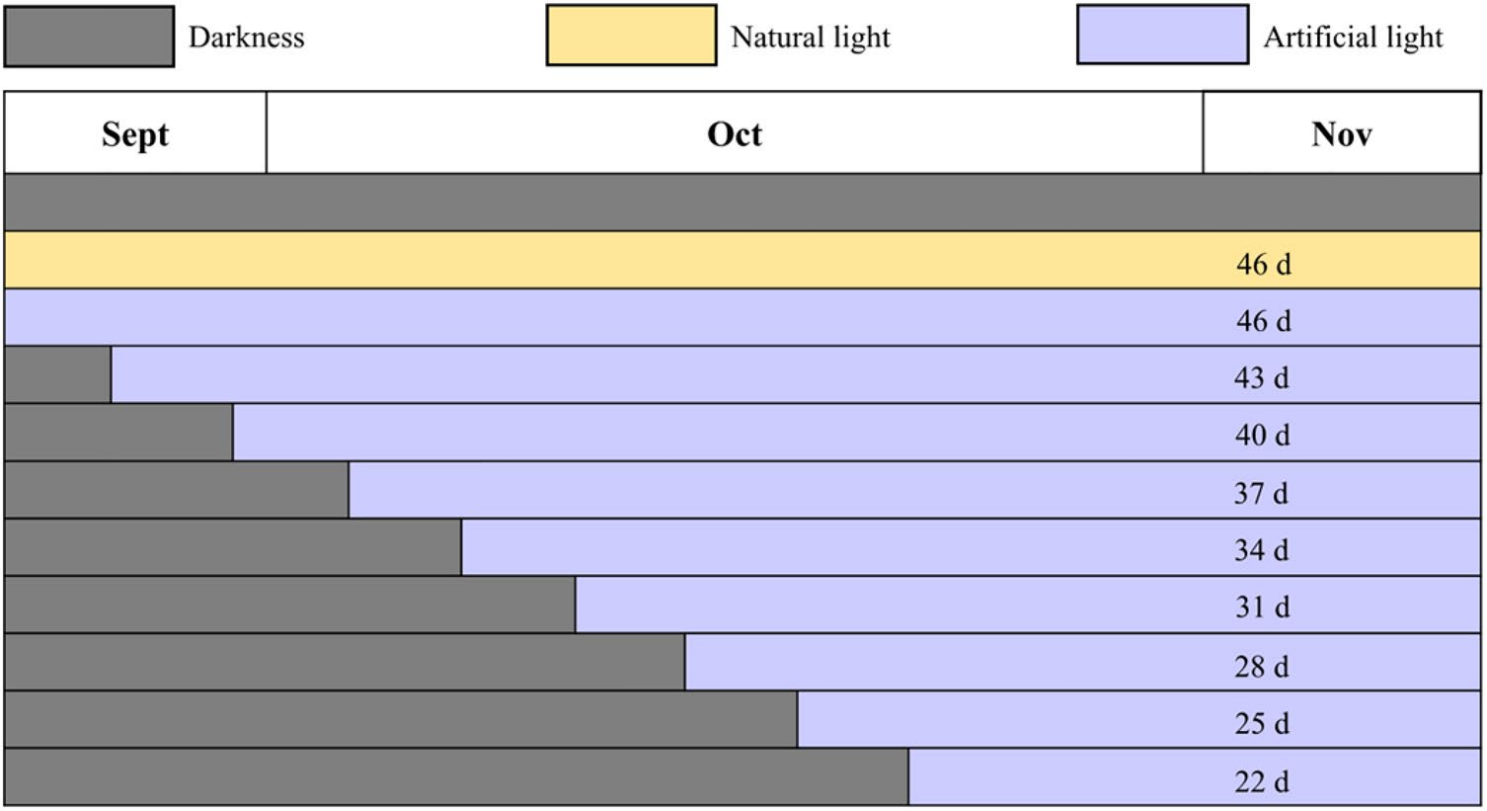
Light exposure days for each treatment.

Saffron corms were obtained from Chongming Island (latitude 121° 40′ N, longitude 31° 62′ E, Shanghai, China). The corms were harvested from the field in mid-May of 2021, dried and sterilized, and then moved indoors for darkness storage. During this period, the corms entered dormancy. The indoor average temperature was set to 28 °C, and the average humidity was controlled to 60–80%. After buds accelerating differentiation and entering in the flower development stage in the middle and late September, corms with a dry weight of 18 ± 1 g were manually selected and placed in trays (0.9 m × 0.6 m, and 0.05-m height) without soil substrate. 200 corms including three replicates as a treatment were processed. The selection range of these corms dry weight conformed to normal distribution principle. The acquisition of saffron corms and preparation before experiments were largely the same as for the experimental methods described by Wang et al.^9^ On September 25th, corms were transferred to indoor cultivation laboratory with 30 m^2^ area and 3 m height, and were exposed to artificial light, natural light, or darkness for treatments from September 26th until blooming, as shown in Fig. 1.

There was a total of eleven treatments for corms, including nine artificial light treatments, an original natural light treatment, and a control dark treatment. They were all placed at the same height of the culture shelf in the lab, and were separated with non-reflective black cloths to avoid interference between treatments. The corms under artificial light treatments were only irradiated the combination of blue and red light 40 cm above the trays. The corms in original natural light treatment were subjected to natural light near the window, which was consistent with the conditions of non-shading treatment in original indoor cultivation. The rest of corms in control treatment were cultivated with shading and darkness. The average temperature of the laboratory was maintained at 18 °C, and the average humidity was set at 70%. In order to ensure smooth air, an air circulation system was employed during the experiments. Except for light conditions, other environmental conditions remained the same.

### Artificial light treatments

**Figure 2:**
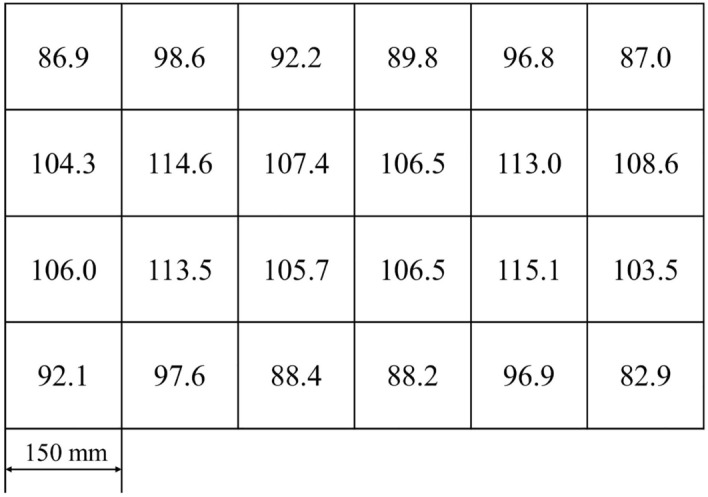
PPFD values of 24 locations on the trays under artificial light treatments with an average PPFD 100.1 μmol m^−2^ s^−1^ and a lighting uniformity 0.83.

**Figure 3:**
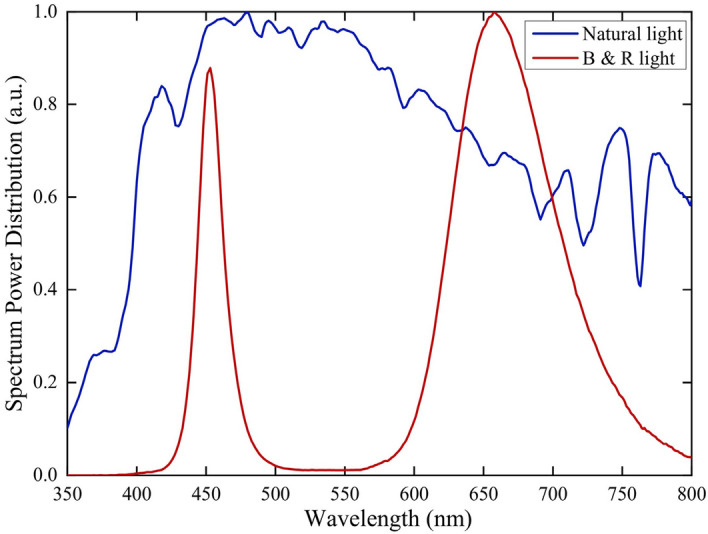
Spectra of different artificial light treatments and original natural light treatment.

Nine luminaires were independently customized according to a single layer size of indoor culture shelf. Every luminaire employed four pieces of blue- and four pieces of red- planar-array light-emitting diode (PA-LED) modules. Each PA-LED module featured 360 pieces of blue LED flip-chips with rated power 0.2 W, peak wavelength 450 nm and full width at half maximum (FWHM) 15 nm. The LED chips were uniformly welded on a ceramic printed circuit board (PCB) and were directly made out a blue PA-LED module by coating with silicone. The additional red PA-LED modules was made out by blue chips coating with the mixture of silicone and red phosphor powder, which has peak wavelength 660 nm and FWHM 85 nm. The red phosphor can fully absorb the blue light emitted by the LED chips. All luminaires were 900 mm *L *× 600 mm *W* and were connected to a dimming controller capable of regulating light output of blue- and red- PA-LED modules. Water-cooling radiators were installed on the back of PA-LED modules, in order to timely export the heat generated by modules and prevent high temperature from affecting corms development and flowering. Nine artificial light treatments were arranged different irradiation days, shown in Fig. 1, and different total daily light integral (TDLI), listed in Table 1. They were set the same photoperiod of 10-h light and 14-h dark with an average photosynthetic photon flux density (PPFD) of 100 μmol m^−2^ s^−1^, following the results provided by Zhu et al.^20^ The PPFD was determined on 24 locations in the trays using a light analyzer for plants (PLA-20, EVERFINE, China), and the measurement results shown in Fig. 2. The measured average PPFD value was 100.1 μmol m^−2^ s^−1^ and the lighting uniformity was 0.83, meeting the uniformity above 0.7 recommended by Radetsky^23^. It was worth noting that the red PA-LED modules could radiant a wide range spectrum from 600 to 800 nm, thus red light also actually included a small amount of far-red light (700–800 nm) in this study. The ratio of B:R:FR = 20%:62%:18% was controlled in the experiments and their relative spectral distributions were shown in Fig. 3. And the ratio was referring to the recommended by Ji et al.^8^ and Zhu et al.^17^.

**Table 1:** Artificial light treatments with different TDLI and other treatments.

No	Treatment	Spectrum	TDLI^a^ (mol m^−2^)
1	Artificial light	B:R:FR = 20%:62%:18%	166
2			155
3			144
4			133
5			122
6			112
7			101
8			90
9			79
10	Original	Natural light	58
11	Control	Darkness	0

^a^TDLI (Total daily light integral) = DLI × days = PPFD × photoperiod × days.

### Determination of the indoor TDLI of natural light

**Figure 4:**
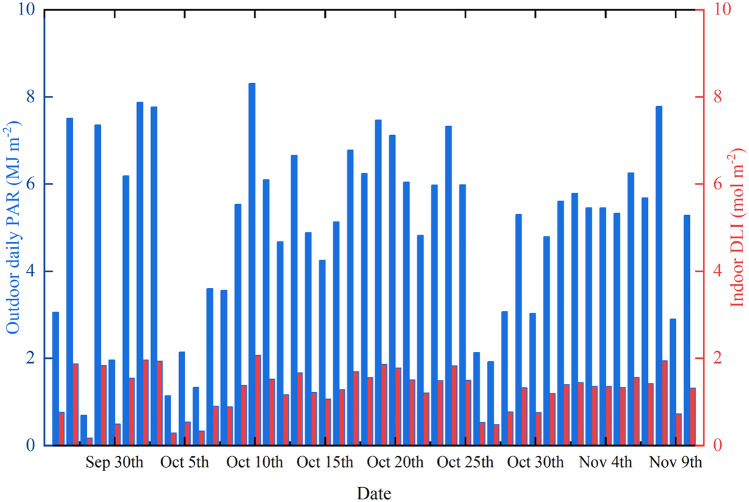
Daily PAR outdoors () and DLI on the trays indoors () under natural light treatment.

During the experiment period, the photosynthetically active radiation (PAR) from natural light was recorded per 10 min by a tinytype meteorological station (YG-GF, YIGU, China), located outside the lab without any shelter. And the outdoor daily PAR was shown in Fig. 4. Besides, selected a sunny noon, the average PPFD in the trays was measured using the light analyzer for plants (PLA-20, EVERFINE, China) under the natural light treatment. Then the ratio of indoor PPFD (μmol m^−2^ s^−1^) and outdoor PAR (W m^−2^) at the same time could be calculated. Then the indoor daily light integral (DLI) received by the corms in the natural light treatment was calculated, shown in Fig. 4. The TDLI from natural light treatment during the experiment period was obtained, listed in Table 1.

### Corm flowering and leaf measurements

Saffron corms in different treatments successively initiated to bloom in early November at the laboratory. Fresh flowers were picked 10 mm below the petals by hand, counted and recorded, and then fresh stigmas were separated manually for counting and recording. The fresh stigma was dried at 65 °C for 40 min in a blast drying oven (DHG-9080A, HUITAI, China) to obtain dry stigma. The flower fresh weight (FW), stigma fresh weight (FW) and dry weight (DW) were measured respectively for three times by an electronic balance (HC3204, HOCHOICE, China). The dry stigmas were stored in closed glass containers and kept in darkness until qualitative analysis. The drying conditions of fresh stigma, and measurement methods of fresh weight or dry weight are following the methodology described by Zhu et al.^17^ The total contents of crocetin esters (including crocetin-I(C_44_H_64_O_24_) and crocetin-II(C_38_H_54_O_19_)), shall not be less than 10.0%, were determined by the spectrophotometry according to the standard ISO 3632-1-2011 previously used in Koocheki et al.^1^ The determination was entrusted to the technical testing center of Shanghai Traditional Chinese Medicine Co., Ltd, and all analyses were carried out in triplicate.

At the end of flowering period, fifteen corms were randomly selected per repetition from each treatment to obtain the morphological characteristics of corm leaves, including leaf length, leaf width and bud length. Leaf length was measured from the tip of the longest leaf to the base of the corm terminal bud. Leaf width was recorded from the middle position of the longest leaf. Bud length was also measured from the top of the gray sheath to the base of the corm terminal bud. Then the leaf area beyond bud could be calculated by leaf width and leaf length exposing the terminal bud, which referred to the green leaf area outside the gray sheath of terminal bud.

### Statistics

Pearson correlation (*P *= 0.01, 0.05) were performed to identify the relationship of TDLI and flower, stigma and leaf parameters using IBM SPSS Statistics software (version 25, USA). A simple liner regression was conducted to analyze the quantitative response to increasing TDLI. Differences among means of leaf parameters were determined by one-way analysis of variances (ANOVA) and the Duncan’s multiple comparison test with significance defined at *P *≤ 0.05.

## Results

### Dependence of corm flowering characteristics on TDLI

**Figure 5:**
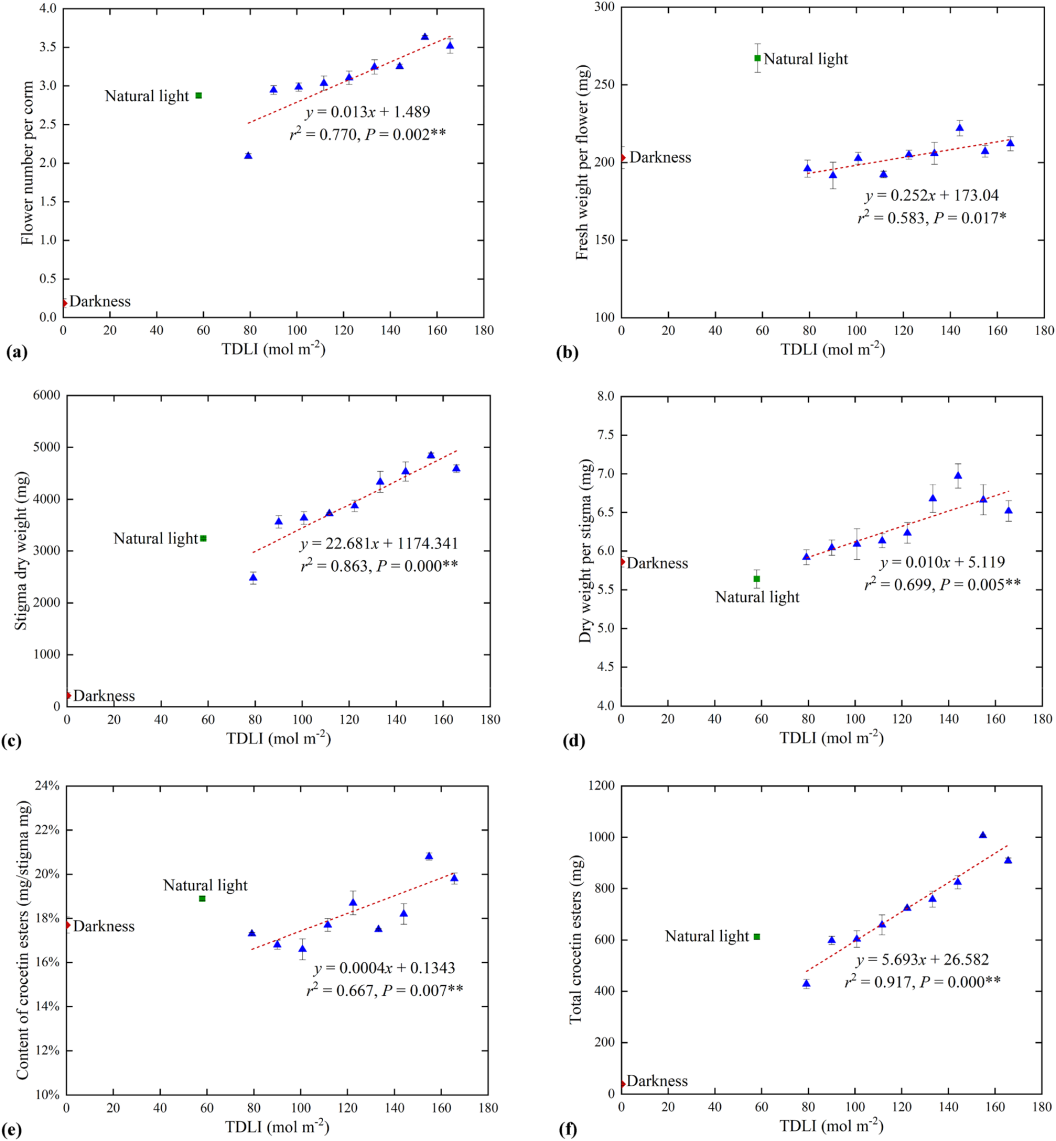
(**a**) Flower number per corm, (**b**) fresh weight (FW) per flower, (**c**) total dry weight of stigma, (**d**) dry weight per stigma, (**e**) content of crocetin esters (contains crocetin-I and crocetin-II) and **(f)** total crocetin esters under different artificial light (), original natural light () as well as control dark treatments (). Mean values ± SE (200 replicate plants). Dashed line represents significant linear regression within each artificial light treatment. ** and * are significant at 1% and 5% probability levels, respectively.

A significant correlation (*P* = 0.002 < 0.01) between flower number per corm and TDLI of blue and red light in artificial light treatments was observed (Fig. 5a). The flower number per corm increased linearly by 73.7% with TDLI increase from 79 to 166 mol m^−2^. It can be inferred that each shoot of corms had an increased possibility of producing two flowers when the TDLI from blue and red irradiation is on the increase. Except for the 79 mol m^−2^ TDLI treatment, the average flower number per corm under other artificial light treatments were greater compared to that under original natural light treatment, with a maximum of 0.75 more flowers. Besides, a single corm only bloomed 0.19 flowers on average under control dark treatment, and the blooming may be caused by turning on the lamp when observing the growth state of corms.

**Figure 6:**
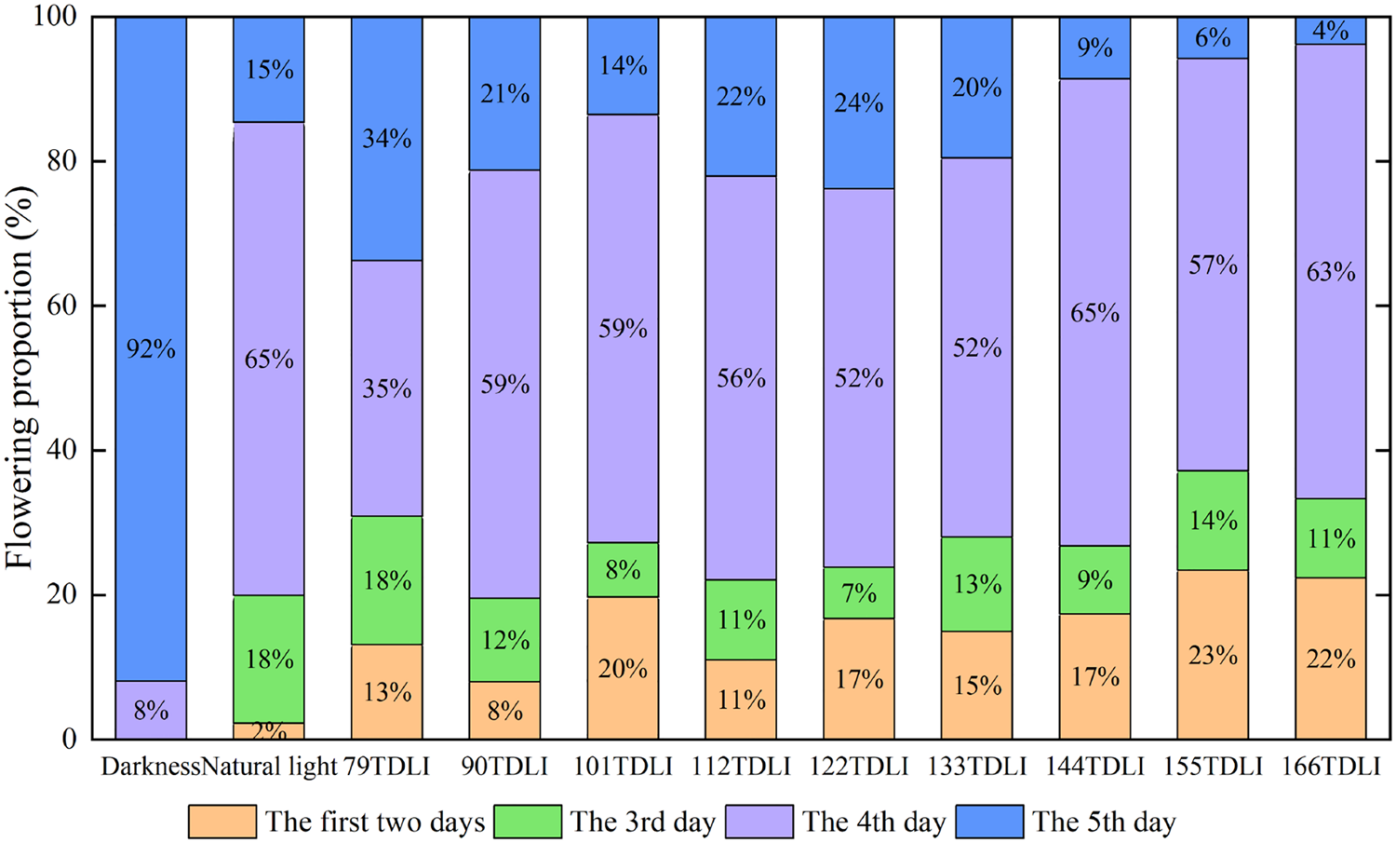
Daily flowering proportion of saffron corm under different artificial light, original natural light as well as control dark treatments. Due to less than 1% of total flower number under few treatments in the first day, flowering proportion was combined in the first two days.

The corm flowering was observed concentrated in each treatment, which lasted for 5 days except for control dark treatment. The flower numbers every day were recorded and divided by total flower numbers (Fig. 6). It was clearly that flowering proportion in the first two days had a positive correlation (*r* = 0.730, *P* = 0.025 < 0.05) with the TDLI of blue and red light. The flowering proportion under TDLI 155 and 166 mol m^−2^ was greater, 193% and 180% of that of TDLI 90 mol m^−2^ in the first two days, respectively. A large number of flowers bloomed on the 4th day, accounting for a high percentage in each treatment except for control dark treatment. The proportion of flowering decreased significantly as TDLI of blue and red light increased (*r* = 0.836, *P* = 0.005 < 0.01) in the fifth day. There was only 3.8% flowering percentage under TDLI 166 mol m^−2^ that day, comparing with 33.7% under TDLI 79 mol m^−2^. Moreover, the flowering duration under original natural light treatment was the same as that under all artificial light treatments, but flowering proportion in the last two days accounted for 80%. The corms treated with darkness delayed flowering for 3 days, which may be caused by the interference of turning on the lamps during observation.

The FW per flower under original natural light treatment was shown greatest, which was 139% of the minimum FW per flower under artificial light treatments (Fig. 5b). The FW per flower had little difference in each artificial light treatment, but there was still a positive correlation (*P* = 0.017 < 0.05) between FW per flower and TDLI of blue and red light. In addition, the FW per flower in control dark treatment was similar to that in artificial light treatments, which further verified the conjecture that a small amount of flowering was caused by interference.

### Dependence of stigma quality characteristics on TDLI

The total stigma DW in each tray increased with the increasing TDLI of blue and red light (*P* = 0.000 < 0.01, Fig. 5c). This trend was similar to flower number, but different from flower FW. Corms had an increasing dry stigma yield up to 96% increment, as TDLI from 79 to 166 mol m^−2^. Except for the TDLI 79 mol m^−2^ treatment, the total stigma DW under other artificial light treatments were higher than that under original natural light treatment. Additionally, only a small fraction of corms bloomed under darkness, so the total stigma yield was very low, close to 0 mg.

The yield per stigma is not related only to total stigma yield, but also flower number, thus DW per stigma is also needed to be analyzed. The DW per stigma increased with the increase of TDLI combined blue and red light under artificial light treatments (*P* = 0.005 < 0.01), up to 6.97 mg at TDLI 144 mol m^−2^, which was 1.24 times of that under original natural light treatment (Fig. 5d). Furthermore, the DW per stigma under original natural light treatment was less than all those under artificial light treatments, while the DW under control dark treatment was close to that under the minimum TDLI treatment from blue and red light irradiation.

The crocetin esters content of dry stigma showed a slight increasing trend with the increase of TDLI by combining blue and red light, but still a significant correlation (*P* = 0.007 < 0.01, Fig. 5e), which all met the commodity requirements of above 10.0%. The corms irradiated under the TDLI 155 and 166 mol m^−2^ treatments had 1.9% and 0.9% higher crocetin esters content than corms under original natural light treatment, respectively, but there was little difference between original natural light treatment and other artificial light treatments. Corms under control dark treatment had a small amount of flowering caused by experimental interference, thereby the crocetin esters content was close to that under several artificial light treatments.

Total crocetin esters followed the trend of total stigma DW under each treatment. The total crocetin esters in artificial light treatments displayed a significant liner response to the TDLI of blue and red light (*P* = 0.000 < 0.01, Fig. 5f), indicating that adding TDLI increased total crocetin esters of saffron in each tray. Comparing with original natural light treatment, corms in each tray can obtain more total crocetin esters up to 394.2 mg, when the TDLI received from the combination of red and blue light was greater than 100.8 mol m^−2^. Corms under darkness had little total crocetin esters due to few flowers.

### Dependence of morphological characteristics of corm leaves on TDLI

**Figure 7:**
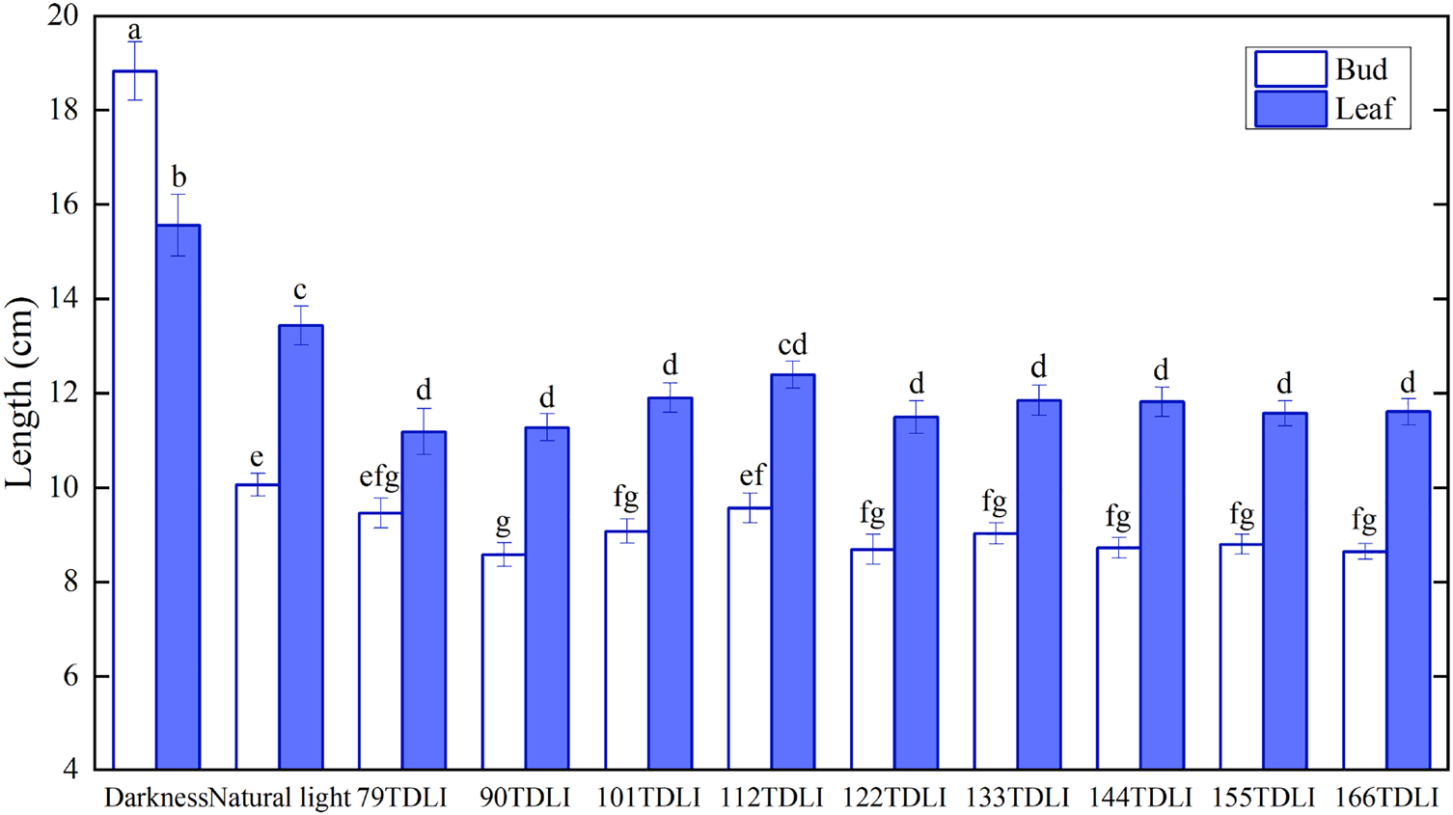
Bud length () and leaf length () under different artificial light, original natural light as well as control dark treatments. Mean values ± SE (15 replicate plants). Different letters in each treatment indicate significant differences based on Duncan’s test (*P* ≤ 0.05).

**Figure 8:**
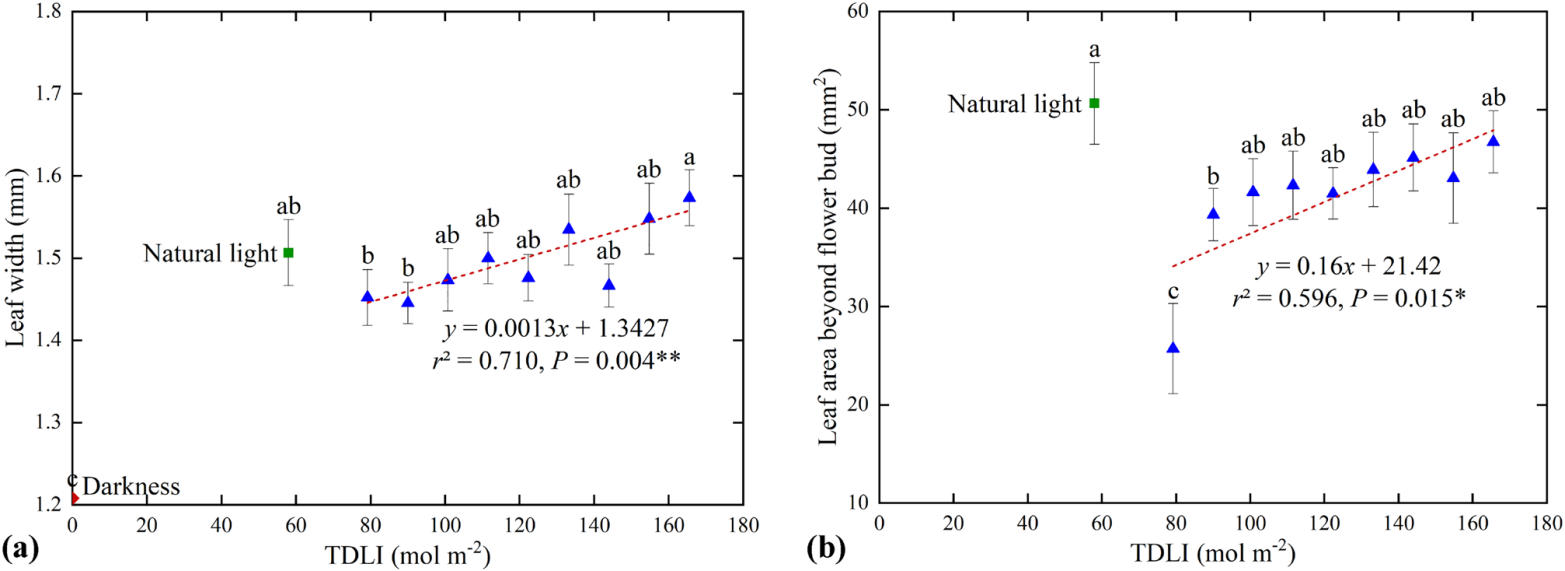
(**a**) Leaf width, and (**b**) Leaf area beyond flower bud under different artificial light (), original natural light () as well as control dark treatments (). Mean values ± SE (15 replicate plants). Different letters in each treatment indicate significant differences based on Duncan’s test (*P* ≤ 0.05). Dashed line represents significant linear regression within each artificial light treatment. ** and * are significant at 1% and 5% probability levels, respectively.

The growth status and morphological characteristics of shoots and leaves under control dark treatment showed a significant difference comparing with those under natural and artificial light treatments (Fig. 7). All leaves in darkness were wrapped and not exposed in the gray sheath of shoots, thereby showed a yellow state with no photosynthesis. The plants had very long shoots, which was 119% longer than the minimum of that under artificial light treatments. Also, the corms in darkness had very long leaf of 15.56 cm, which was 1.4 times of the minimum one with artificial light treatments. On the contrary, the corms under control dark treatment had the narrowest leaf of 1.21 mm, which was 77% of the maximum one with artificial light treatments (Fig. 8a). It was indicated that most of nutrients provided by saffron corms were used to supply the growth of shoots and leaves in absence of light. In addition, the morphology of shoots and leaves under original natural light treatment was similar to that under artificial light treatments. All leaves rushed out of the gray sheath of shoots and exposed some green leaves. The leaf length under original natural light treatment showed longer compared with those under artificial light treatments, up to 13.43 cm, but no great difference in leaf width (Figs. 7, 8a). Besides, no significant differences in bud length and leaf length were found among any artificial light treatments (*P* = 0.186, 0.551).

The TDLI of combining blue and red light had positive correlation with leaf width of corms (*P* = 0.004 < 0.01, Fig. 8a). There was a slow increase tendency for leaf width to increase with TDLI (*r*^2^ = 0.710). The leaves under the TDLI 166 mol m^−2^ treatment were the widest, up to 1.57 mm, but only a minor change of 8% compared to that under the TDLI 79 mol m^−2^ treatment. There were no significant differences in leaf width among artificial light and natural light treatment.

Since part leaf area at the corm base was covered by the gray sheath of shoots, leaf area beyond bud was calculated so as to further explore leaf photosynthesis. The leaf area beyond bud was positively (*P* = 0.015 < 0.05) affected by increase in the TDLI of blue and red light (Fig. 8b), indicating TDLI affected the extent to which leaves rushed out of the gray sheath of shoots. Low leaf area beyond bud was observed for corms under less than TDLI 90 mol m^−2^. The leaf area exposed shoot under the TDLI 166 mol m^−2^ treatment was 46.73 mm^2^, which was 1.8 times of that with TDLI 79 mol m^−2^ treatment. In addition, the largest leaf area beyond bud was under original natural light treatment, up to 50.64 mm^2^, about twice of that with TDLI 79 mol m^−2^ treatment. It should be noted that the leaves under the darkness did not expose shoots, thus the data was not displayed in Fig. 8b.

## Discussion

To our knowledge, there have been little previous studies investigating the effect of artificial lighting on indoor saffron corm without any cultivations by soil^1,4,6,24^, sand^5^, nutrient solution^13^ and other substrates^16,22^, but our results about flowering and leaves are similar to those described for other plants. Plant flowering is a complex phenomenon where flower formation duration, flower number and flower size are particularly regulated by photosynthetic photon flux density (PPFD), light duration and light spectrum^25^. There has been confirmed that an increment in daily light integral (DLI), either by increasing the PPFD with the same photoperiod, or by extending the photoperiod at the same PPFD, promoted growth and flowering of cyclamen^26^. Lee et al*.*^27^ showed that increasing DLI promoted flower initiation and the number of inflorescences and accelerated spiking of *Phalaenopsis* plants. Llewellyn et al*.*^28^ reported that cumulative flower production increased linearly as DLI increase, and doubling the total DLI from 6 to 12 mol m^−2^ d^−1^ by providing the supplemental PAR from LEDs could increase the number of flowers produced by nine flowers per ‘Panama’ plant.

In the present study, the increase in TDLI received from blue and red light significantly promoted flower number and also affected flowering duration (Figs. 5a, 6). The corms under the 79 mol m^−2^ TDLI had the smallest number of flowers, only 2.09 per corm, which was remarkably lower than that under other artificial light treatments. There might be mostly a threshold between 79 and 90 mol m^−2^ TDLI beyond which the flower number increases significantly. The flowers under natural light treatment had a greatest FW (Fig. 5b), but flower number per corm was only greater than that under the 79 mol m^−2^ TDLI treatment (Fig. 5a). Therefore, from the perspective of flower number, once the TDLI received from the combination of red and blue light exceeds 90 mol m^−2^, it will probably replace original indoor natural light treatment.

It has been reported that corm size plays an important role in achieving the optimal yield of saffron. The heavier mother corms, the higher percentage of flower emergence and yield of saffron^3,29^. Khorramdel et al*.*^24^ demonstrated that 5–10 g mother corms and 15% concentration of foliar fertilizer were the most favorable condition to reach the highest number of flowers (38.63 m^−2^). There was an increment in total flower number of saffron per square meter when medium-sized mother corms were compared to small-sized corms^24,30^. Cardone et al*.*^31^ reported that the corms with a dimension of 3.6–4.5 cm and a mean weigh of 25 g planted in annual crop cycle, produced the highest dry weight of stigmas (7.4 mg). In the present study, corm development and flowering only depended on the regulation of environmental factors during indoor cultivation, which were different from those described above that required the soil, water, and fertilizer, etc. Hence the emergence, development and maturity of flowers were inseparable from the carbohydrates stored by corms, and flower number had an extremely strong correlation with the corm size^4^. The ability of four flowers produced per corm in our experiments were subjected to the medium-sized corms, but flower number was increasing with the increase of the TDLI up to 150 mol m^−2^ by artificial lighting. It has been reported that flower numbers increase as DLI increases until some threshold beyond which a further increase has little or no effect on developmental rate^26,32^. Therefore, the TDLI 150 mol m^−2^ from blue (peak wavelength 450 nm, FWHM 15 nm) and red (peak wavelength 660 nm, FWHM 85 nm) LEDs may be an appropriate artificial lighting scheme.

Furthermore, flowering time was not obviously reflected in advance or delay of the days to first flower in this study, but the daily flowering proportion in total flower number. The flowering proportion in the first two days increased and decreased on the 5th day with the increasing TDLI of blue and red light. Phytochrome mainly sense red (600–700 nm) and far-red (700–800 nm) radiation, and cryptochromes primarily absorb blue (400–500 nm) radiation, which are both known to coordinately regulate plant morphological and developmental traits, including stem elongation, chlorophyll synthesis, and flowering time^24,33^. Yoshida et al*.*^34^ reported that blue LEDs with a peak wavelength of 450 nm promoted flowering compared to red LEDs with a peak wavelength of 660 nm. Wollaeger et al*.*^35^ found that the number of shoots of impatiens (*Impatiens walleriana*) increased as the portion of blue radiation increased from 0 to 100%. Nevertheless, Park et al*.*^33^ provided that a moderately high blue photon flux density (B:R = 1:1) attenuated the effects of the R:FR ratio on extension growth but had no apparent effect on the FR promotion of flowering in a long-day plant, and decreasing the ratio of R:FR promoted subsequent flowering by 7–11 d. As shown in the above researches, the response of flowering time on blue and red LEDs is variety dependent. In the present study, the same flower duration is likely attributed to the same ratio of blue and red light, while different flowering proportion every day may be stimulated by different TDLI from artificial lighting. The flowering duration under original treatment lasted the same with the light treatments, but flowering proportion in the first two days was much lower than those in all light treatments, accounting for only 2.3% (Fig. 6). This further verified that the daily flowering proportion was more affected by TDLI than light spectrum. Different DLI had only minor effects on flower FW of ‘*Panama*’^28^, which was similar to our results. In the present study, flower FW had only increased slightly under different TDLI of blue and red LEDs (Fig. 5b).

Since one saffron flower can normally produce three stigmas, total stigma yield in saffron is highly correlated with flower number^6,36,37^, which is in agreement with our results. In accordance with the flower number, the increasing TDLI from blue and red light promoted significantly total stigma DW, which were greater than that under original natural light treatment except for the TDLI 79 mol m^−2^ treatment (Fig. 5c). Obviously, after corms entered stigma differentiation, the original indoor natural light treatment can be replaced by TDLI higher than 90 mol m^−2^ combing blue with a broad-band red LEDs so as to increase dry stigma yield, and up to 1.5 times increment. Furthermore, in our experiments, DW per stigma under all mixed blue and red light treatments were significantly higher than that of original natural light treatment (Fig. 5d), which further indicated that combining blue and red light was conducive to the increase of yield per stigma.

Crocetin esters, as the main active compounds in saffron, are responsible for its coloring power^1,5,19,21,22^, hence their content has been utilized to evaluate stigma quality. Gresta et al*.*^36^ concluded that colder environments reduced the amount of crocetin esters and consequently lower stigmas quality. In the present study, higher TDLI by blue and red LEDs radiation increased slightly the content of crocetin esters (Fig. 5e). Considering the total stigma yield, the total crocetin esters was the largest under 150 mol m^−2^ TDLI treatment, which was significantly higher than that in the original natural light treatment (Fig. 5f). Therefore, it can be further concluded that TDLI 150 mol m^−2^ from blue and a broad-band red LEDs might obtain excellent stigma quality.

Different from other monocot plants, indoor cultivation of saffron corm in this study primarily goes through the process including shoot differentiation, flower incubation and leaf growth and flowering. The flower and leaf developments occur in shoots, and then grow out of the shoots. Once the long scarlet stigmas are observed, the flower has been present mature and can be picked. The part leaves wrapped in the gray sheath of shoots can hardly receive light and thus are unable to carry out photosynthesis. Hence the study of leaf growth morphology should be focused on the leaf area beyond buds. He et al*.*^38^ found that all leaves of sweet potato (*I. batatas*) had similar area and water content under different TDLI by a combination of blue (463.5 nm) LEDs and red (633 nm and 656 nm) LEDs in the ratio of 9:1. Huber et al*.*^39^ investigated that the total leaf area per plant in tomato seedings was not affected by increases in the DLI. Both these two investigations were close to our results of leaf area beyond buds. In this study, the increasing TDLI from blue and red light had a slight increase in leaf area beyond buds (Fig. 8b). It was likely that the majority corm leaves were still wrapped in the gray sheath of shoots, and they were not yet mature, and had weak photosynthesis. Hence the increasing TDLI received from blue and red light combination did not have a very positive effect on the increase of leaf area beyond buds and carbohydrate formations. On the contrary, the flowers in the gray sheath of shoots were in a critical period of flower development at the time. The TDLI increase of blue and red light was contributed to the development and maturity of the flowers, which promoted the increment in flower number, and increased the probability of young flowers and small flowers to bloom out of the gray sheath of shoots. Accordingly, there was no significant difference of leaf area beyond buds among light treatments except for the 79 mol m^−2^ TDLI treatment. Leave growth mainly occurred in the vegetative development during winter and early spring months, which can supply primary carbohydrates for the various plant sinks by photosynthetic activity^4^. The leaf area beyond bud under the 79 mol m^−2^ TDLI was significantly less than that under other artificial light treatments. It can be verified again that the previous conjecture about a threshold between 79 and 90 mol m^−2^ TDLI beyond which the flower number and leaf area increases significantly.

The leaf area is also influenced by light spectrum, but requires specific species consideration. When plants receive the combination light emitted by blue and red LEDs, leaf area decreased with the increase of blue PPF (*P* < 0.0001) in cucumber^15^. In saffron plants, leaf DW and leaf length increased under monochromatic red LEDs compared to those under monochromatic blue LEDs. Morphological characteristics of leaves had a tendency under different ratios of blue and red light, but still had not been found regular^16^. In the present study, comparing to the minimum light treatment of 79 mol m^−2^ TDLI, both of the shoot and leaf were longer under the natural light treatment (Fig. 7), and leaf width had no significant difference (Fig. 8a), and thereafter the leaf area beyond bud was greater (Fig. 8b). However, the leaf area beyond bud under the natural light treatment had no significant differences with other artificial light treatments. Thus, the saffron leaves had a weak photosynthesis in the early stage of development for the indoor saffron corm cultivations, and the relationship between morphological characteristics of corm tender leaves and light spectrum had not been verified.

Last but not least, once the TDLI from blue and red light exceeded 90 mol m^-2^, the flowering number and stigma yield were more than those under natural light. However, it had to be admitted that the TDLI of natural light was lower than artificial light, only 58 mol m^-2^. The natural light has rich spectral species, especially contains a low proportion of red and far-red light, which can be perceived by phytochromes and has a remarkable effect on germination, flowering and photosynthesis^40^. Asuka et al*.*^41^ investigated that flower initiation and induction of *Eustoma grandiflorum* ‘Nail Peach Neo’ were promoted by night break treatment with a low R:FR irradiation, but was delayed by a high R:FR ratio. The promotion or delay of flower bud formation was accompanied by a decrease or an increase, respectively, in the number of nodes on the main stem at anthesis to the first floret. Felix et al*.*^42^ found that far-red light could advance flowering by 10 and 20 days in some amaranth and rice genotypes, respectively, but have no impact on flowering in soybeans. In our experiments, there existed a lower R:FR of 54%:46% under natural light treatments than that in artificial light treatments. The high proportion of far-red light in natural light promoted the flowering number (Fig. 5a) to a certain extent, and thus increased the stigma yield (Fig. 8). And the highest flower FW (Fig. 5b) was noted under the natural light treatments with significant differences with all artificial light treatments.

In addition, green light in natural light also plays a vital role in photosynthesis, as it helps plants to adapt to different light intensities. Compared to blue and red light, green light can deeper into leaves, resulting in more uniform light distribution throughout leaves^43^. In the present study, the saffron leaves were not fully developed and had a weak photosynthesis, so there was no significant difference of the leaf area beyond bud between natural light and artificial light treatments (Fig. 8b). Nevertheless, the leaf length and leaf area beyond bud at low TDLI under indoor natural light had exceeded those at the highest TDLI mixed by red and blue light, which was more conductive to the subsequent leaf photosynthesis and corms propagation after entering the field growth stage.

In order to remain the same light condition under original treatment as the original indoor cultivation, the DLI indoors of natural light in our experiments was changeable every day (Fig. 4) and uncontrollable. Therefore, if artificial simulation of natural light is considered to irradiant the saffron corms by effectively controlling, the flowering number and stigma yield may be further increased, and the larger leaf area can also help photosynthesis during the field cultivations.

## Conclusions

Regarding to the results and discussion above, TDLI received from the combination of blue and broad-band red LEDs had significant effects on flowering characteristics and stigma yield of saffron. Compared with the original indoor cultivation of natural light, the TDLI above 90 mol m^−2^ mixed by blue LEDs (peak wavelength 450 nm, FWHM 15 nm) and red LEDs (peak wavelength 660 nm, FWHM 85 nm) can accelerate flowering process, promote flower number, and improve stigma yield at the cost of flower FW reduction. Flower number per corm and stigma yield were highest in the TDLI 150 mol m^−2^ treatment, which can be considered as an optimal artificial lighting condition during indoor saffron cultivation.

Light plays a crucial role in shoot differentiation, flower incubation, leaf growth and flowering of saffron. For indoor corms cultivation with darkness treatment, the saffron are unable to bloom because there is no light modulation to shoots and leaves. Low proportion of R:FR in natural light contributes to flowering and stigmas yield in increase, and green light also plays a vital role in leaf photosynthesis. Moreover, the increasing TDLI from blue and red LEDs could slightly promote leaf width and area beyond buds, but had no significant effect on bud length and leaf length.

## Data Availability

The data presented in this study are available on request from the corresponding authors.
